# Bibliographical

**Published:** 1888-09

**Authors:** 


### BIBLIOGRAPHICAL.

THE PHYSICIAN'S LEISURE LIBRARY, NUMBER 7. The Modern Treatment of Pleurisy and Pneumonia. By G. M. GARLAND, M. D.; Numbers 8 and 9. The Infectious Diseases. By KARL LIEBERMEISTER. Translated by E. P. HURD, M. D.

These are consecutive numbers of a series of medical works issued monthly by Geo. S. Davis, medical publisher, of Detroit, Mich. Subscription price $2.50 per annum, 25 cents per number.

TRANSACTIONS OF THE NEW YORK ODONTOLOGICAL SOCIETY FOR 1887. Philadelphia: The S. S. White Dental Manuf'g Co.

This volume is uniform with the volumes of the transactions for some years past. It contains some valuable papers, and the cream of the discussions for the year, and forms another number of a valuable series. Of course it is handsomely printed and bound, for the S. S. White Dental M'fg Co. attended to that.

TRANSACTIONS OF THE DENTAL SOCIETY OF THE STATE OF NEW YORK FOR THE YEARS 1879, 1880 AND 1881.

These are the long lost records, which for so many years have been buried——somewhere. Even now, some of the most valuable papers are irretrievably gone. The matter was excellent in its day, but it has "an ancient and a fish-like smell" now, and there was no excuse for its publication, save the important one of completing the history of the society.

